# Plant Oils as Potential Sources of Vitamin D

**DOI:** 10.3389/fnut.2016.00029

**Published:** 2016-08-12

**Authors:** Anja C. Baur, Corinna Brandsch, Bettina König, Frank Hirche, Gabriele I. Stangl

**Affiliations:** ^1^Institute of Agricultural and Nutritional Sciences, Martin Luther University Halle-Wittenberg, Halle (Saale), Germany

**Keywords:** ergosterol, 7-dehydrocholesterol, vitamin D, plant oils, wheat germ oil, ultraviolet light irradiation, bioavailability, mice

## Abstract

To combat vitamin D insufficiency in a population, reliable diet sources of vitamin D are required. The recommendations to consume more oily fish and the use of UVB-treated yeast are already applied strategies to address vitamin D insufficiency. This study aimed to elucidate the suitability of plant oils as an alternative vitamin D source. Therefore, plant oils that are commonly used in human nutrition were first analyzed for their content of vitamin D precursors and metabolites. Second, selected oils were exposed to a short-term UVB irradiation to stimulate the synthesis of vitamin D. Finally, to elucidate the efficacy of plant-derived vitamin D to improve the vitamin D status, we fed UVB-exposed wheat germ oil (WGO) for 4 weeks to mice and compared them with mice that received non-exposed or vitamin D_3_ supplemented WGO. Sterol analysis revealed that the selected plant oils contained high amounts of not only ergosterol but also 7-dehydrocholesterol (7-DHC), with the highest concentrations found in WGO. Exposure to UVB irradiation resulted in a partial conversion of ergosterol and 7-DHC to vitamin D_2_ and D_3_ in these oils. Mice fed the UVB-exposed WGO were able to improve their vitamin D status as shown by the rise in the plasma concentration of 25-hydroxyvitamin D [25(OH)D] and the liver content of vitamin D compared with mice fed the non-exposed oil. However, the plasma concentration of 25(OH)D of mice fed the UVB-treated oil did not reach the values observed in the group fed the D_3_ supplemented oil. It was striking that the intake of the UVB-exposed oil resulted in distinct accumulation of vitamin D_2_ in the livers of these mice. In conclusion, plant oils, in particular WGO, contain considerable amounts of vitamin D precursors which can be converted to vitamin D *via* UVB exposure. However, the UVB-exposed WGO was less effective to improve the 25(OH)D plasma concentration than a supplementation with vitamin D_3_.

## Introduction

Food sources of vitamin D are scarce. Although oily fish is considered to be a good source of vitamin D_3_ (1, 2), its consumption and its vitamin D content is not high enough to significantly improve the vitamin D status of humans (3). Besides fish, mushrooms are often considered as another valuable source of vitamin D, in particular of vitamin D_2_. However, the major natural vitamin D metabolite in fungi and yeast is the vitamin D precursor ergosterol, which can be converted to vitamin D_2_ by UVB irradiation (4). The UVB-exposed baker’s yeast, which has been approved by the European Food Safety Authority as a reliable ingredient to enrich bakery products with vitamin D, is a prominent example for a successful application of UVB irradiation to enhance vitamin D in natural foods (5). However, less data are available on vitamin D precursors and metabolites in plants. Yellow oat grass (*Trisetum flavescens*) is well described for its capability to synthesize bioactive vitamin D. It contains vitamin D glycosides which can be hydrolyzed in the gut or by the gastrointestinal microflora to the biologically active 1,25-dihydroxyvitamin D (6–8). Other so-called calcinogenic plants that contain active vitamin D forms are *Solanum malacoxylon, Cestrum diurnum*, and *Nierembergia veitchii* of the *Solanaceae* family (6–8). These plants are presumed to cause calcinosis in grazing animals due to the hypercalcemic effect of toxic 1,25-dihydroxyvitamin D levels (9). Vitamin D metabolites were also found in *Cucurbitaceae, Fabaceae*, and *Poaceae* (10–12). Besides that, certain plants are associated with fungal endophytes (13, 14) or are capable to produce the vitamin D_3_ precursor 7-dehydrocholesterol (7-DHC) on its own *via* the lanosterol pathway (15). Based on these data, we hypothesized that plant oils could also contain vitamin D precursors or metabolites. The main aims of this investigation were [1] to identify and quantify precursors and metabolites of vitamin D in plant oils that are used in human nutrition and [2] to investigate whether a short-term exposure of selected oils to UVB light could increase their vitamin D content. To elucidate possible adverse effects of UVB exposure on the quality of the oils, we analyzed oxidative biomarkers and tested the sensory quality of the UVB-exposed oils. Additional tests were conducted to assess the stability of these vitamin D metabolites subsequent to thermal treatment and storage of the UVB-exposed oil. Finally, we aimed to elucidate the efficacy of plant-derived vitamin D to improve the vitamin D status by feeding an UVB-exposed plant oil to mice.

## Materials and Methods

### Characterization of Vitamin D Metabolites in the Plant Oils

Avocado oil, linseed oil, olive oil, pumpkinseed oil, rapeseed oil, soya oil, sunflower oil, and wheat germ oil (WGO) were used to characterize and quantify their vitamin D precursors and metabolites. From each type of oil, three commercially available representatives were obtained from local supermarkets and used for the analyses. The oil samples selected for analyses were flushed with N_2_ after the first opening, to avoid oxidation processes and stored at 4°C until further analyses.

#### UVB Exposure of Selected Oils

Rapeseed oil, avocado oil, and WGO were used for the UVB treatments and exposed to UVB light. In the first approach, aliquots of the three oils were placed into plastic vessels (thickness of the oil layer 1.0 mm) and exposed to UVB light for 0 (control), 4, and 8 min at room temperature. The UVB-emitting lamp (650 μW/cm_2_, in a distance of 15 cm, UV-8M, Heroloab GmbH, Wiesloch, Germany) was placed 10 cm above the oil surface. During that treatment, the oils were flushed with N_2_. In a second approach, WGO was used to investigate the impact of the oil layer thickness on the efficacy of vitamin D formation through UVB irradiation. Therefore, different volumes of the oil were filled in glass vessels to reach a layer thickness of either 1.6 or 3.2 mm, to be UVB-exposed for 10 min at room temperature. During that time, the oil samples were constantly stirred by a magnetic stirrer under N_2_. The oil samples were stored at −20°C until analyses of vitamin D_2_, vitamin D_3_, and tocopherols. In addition to that, the peroxide and the acid values were analyzed in the 10 min UVB-exposed WGO and compared with those of the non-exposed oil of the same batch. The analyses were complemented by organoleptic tests. The UVB-treated oil (exposure time: 10 min, oil layer thickness: 3.2 mm) which was intended for use in the mouse study was analyzed for vitamin D metabolites and stored at −20°C until preparation of the diet. All diets were stored at −20°C until their administration.

#### Thermal Treatment and Storage of UVB-Exposed Wheat Germ Oil

To estimate the stability of the UVB-exposed oils, aliquots of the 10-min UVB-exposed WGO (1.6 mm layer) were (1) heated at 100 or 180°C for 10 min and (2) stored for 1 day, 2 weeks, or 4 weeks at room temperature in the dark. After the thermal treatment and storage terms, the WGOs were flushed with N_2_ and stored at −20°C until further analysis. Aliquots of untreated oil samples of the same batch were used as a reference. Besides the concentration of the vitamin D metabolites, the concentration of tocopherols were analyzed to gain information about oxidation processes. The thermally treated and the 4 weeks-stored WGOs were analyzed to record the peroxide and the acid values and subjected to organoleptic tests.

#### Analysis of Vitamin D Metabolites in Oils

The concentrations of ergosterol, 7-DHC, vitamin D_2_, and vitamin D_3_ were analyzed by high performance liquid chromatography (HPLC) coupled with a tandem mass spectrometry system (LC-MS/MS), as described elsewhere (16). In brief, aliquots of the oils (300 mg) were mixed with internal deuterated standards (7-DHC-d_6_, Chemaphor Incorporation, Ottawa, ON, Canada; vitamin D_2_–d_3_, vitamin D_3_–d_3_, Sigma Aldrich Chemie GmbH, Taufkirchen, Germany). After an overnight hydrolysis, the D vitamers were extracted with *n*-hexane, washed with ultrapure water, solved in *n*-hexane/isopropanol (99/1, v/v), and fractionated using an Agilent 1100 HPLC-System with a LiChrospher Si 60 column (250 mm × 4.0 mm, 5 μm particle size; Agilent Technologies, Waldbronn, Germany) (17). Subsequent to drying the fractions, 100 μl of 4-phenyl-1,2,4-triazoline-3,5-dione (0.15 mg/ml acetonitrile, Sigma Aldrich) were added for derivatization (18). After evaporation of the solvent, the samples were resolved in methanol, mixed with 10 mM ammonium formate solution (4/1, v/v), and analyzed using HPLC (Agilent 1100) with a Hypersil ODS-column (150 mm × 2.0 mm, 5 μm particle size; VDS Optilab Chromatographie Technik GmbH, Berlin, Germany) coupled with a MS system (API 2000, Sciex, Darmstadt, Germany). Two mixtures with a gradient flow were used as mobile phase (A: 1 mM methylamine in acetonitrile; B: 1 mM methylamine and 5 mM ammonium formate in acetonitrile/ultrapure water, 1/1, v/v). External standards (ergosterol, 7-DHC, vitamin D_2_, vitamin D_3_, Sigma Aldrich) were used for calibration. The multiple reaction monitoring (MRM) was used for quantification at the following mass transitions: ergosterol, 603 > 377; 7-DHC, 591 > 365; 7-DHC-d_6_, 597 > 371; vitamin D_2_, 603 > 298; vitamin D_2_–d_3_, 606 > 301; vitamin D_3_, 591 > 298 and vitamin D_3_–d_3_, 594 > 301.

Except for the analysis of the vitamin D metabolites in rapeseed, avocado, and WGO after UVB exposure (1.0 mm layer), all analyzes were run in duplicate. The lower limit of quantification (LLOQ, signal-to-noise ratio = 10) in oils was 4.3 ng/g for all vitamin D metabolites. The coefficients of variation were 10.1% for ergosterol, 12.8% for 7-DHC, 5.9% for vitamin D_2_, and 9.2% for vitamin D_3_ (*n* = 10).

#### Analysis of Autoxidation Markers in Oils

The concentrations of the tocopherols were measured by a modified HPLC method of Coors (19). Prior to the quantification of the tocopherols, aliquots of the oils were solved in *n*-hexane (1/100, w/v), mixed thoroughly, and separated isocratically by HPLC (Agilent 1100) using a LiChrospher Si 60 column (250 mm × 4.0 mm, 5 μm particle size; Agilent Technologies). A mixture of *n*-hexane and isopropanol (99/1, v/v) was used as mobile phase (flow rate: 1 ml/min). The α-, β-, and γ-tocopherols were detected by a fluorescent detector (emission: 330 nm, excitation: 295 nm). External standards (α-, β-, γ-tocopherols, Supelco, Bellefonte, PA, USA) were used for calibration. The peroxide and the acid values of the oils were determined according to the German official methods (20, 21).

#### Organoleptic Characterization of the Oils

The UVB-exposed, the thermally treated, the 4 weeks-stored, and the -untreated WGOs were evaluated by a trained panel (ÖHMI Analytik GmbH, Magdeburg, Germany) in a blinded fashion. Taste, aroma, color, and transparency of the oils were judged at 40°C (22), and the oils were ranked according to its organoleptic quality (23).

### Mouse Study

The experimental procedures described below followed the established guidelines for the care and handling of laboratory animals according to the National Research Council (24) and were approved by the local government (Landesverwaltungsamt Sachsen-Anhalt, Germany; approval number 42502-5-34). All mice were housed in pairs on a 12-h light, 12-h dark cycle in a room controlled for temperature (22 ± 2°C) and relative humidity (50–60%). Food and water were provided *ad libitum*.

Forty-two 4-week-old male mice (C57BL/6NCrl, Charles River Laboratories, Sulzfeld, Germany) were used. Five weeks prior to the actual treatment, the mice received a vitamin D-free semi-synthetic basal diet (20% casein, 20% sucrose, 38.8% starch, 10% WGO, 6% vitamin-mineral-mixture, 5% cellulose, and 0.2% dl-methionine) to reduce their vitamin D status. Except for the vitamin D, all other vitamins and minerals were supplemented according to the recommendations of the AIN (25). After the 5-week, six mice were sacrificed to determine the vitamin D status of these animals at baseline. The remaining 36 mice (mean body weight: 13.9 ± 0.8 g) were allotted to 3 groups of 12 mice each and fed the basal vitamin D-free diet with 10% of either the 10 min UVB-exposed WGO (3.2 mm-layer, WGO-UV), the untreated WGO, or WGO that was supplemented with synthetic vitamin D_3_ (WGO-D_3_) in comparable amounts to the total vitamin D content analyzed in the UVB-exposed oil. In the experimental diet fed to the WGO-UV group, a mean vitamin D_2_ concentration of 87.3 μg/kg diet was measured, whereas no vitamin D_3_ was found. The diet fed to the WGO-D_3_ group had a mean analyzed vitamin D_3_ concentration of 80.0 μg/kg and no vitamin D_2_, while in the diet fed to the WGO group neither vitamin D_2_ nor vitamin D_3_ could be detected. The experimental diets were fed to the mice for 4 weeks. Individual body weights and mean food intake per cage were recorded weekly. Finally, the mice were sacrificed after a 4-h food deprivation under light anesthesia with diethyl ether. Blood was collected into heparin tubes (Sarstedt, Nümbrecht, Germany). Plasma was separated by centrifugation at 3000 × *g* at 4°C for 20 min and stored at −20°C until analysis of the vitamin D metabolites. The livers were harvested, immediately snap-frozen in liquid N_2_, and stored at −80°C until analysis of the vitamin D metabolites.

#### Analysis of Vitamin D Metabolites in Plasma, Diet, and Liver

The plasma concentrations of ergosterol, 7-DHC, vitamin D_2_, vitamin D_3_, 25-hydroxyvitamin D [25(OH)D], in particular 25(OH)D_2_ and 25(OH)D_3_, were measured using LC-MS/MS (16). Plasma aliquots (100 μl) were mixed with potassium hydroxide, ascorbic acid, sodium sulfide, and internal standards (7-DHC-d_6_, vitamin D_2_–d_3_, vitamin D_3_–d_3_, and 25(OH)D_3_–d_6_, Chemaphor Incorporation) and flushed with N_2_. After incubation for 3 h at 37°C, followed by 17 h incubation at room temperature, the D vitamers were extracted two times with *n*-hexane and mixed with 4-phenyl-1,2,4-triazoline-3,5-dione for derivatization. Subsequently, the extracts were dried, resolved in methanol and 10 mM ammonium formate (4/1, v/v), and analyzed *via* LC-MS/MS as described before for the oils samples. Mass transitions for the hydroxylated metabolites were 25(OH)D_2_, 619 > 298; 25(OH)D_3_, 607 > 298; and 25(OH)D_3_-d_6_, 613 > 298. In plasma samples, the LLOQ of ergosterol, vitamin D_2_, and vitamin D_3_ was 1.25 nmol/l, that of 25(OH)D_2_ and 25(OH)D_3_ was 4.2 nmol/l.

The vitamin D_2_ and D_3_ concentrations in the diets and liver samples were analyzed as already described for the oil samples. The vitamin D_2_ and vitamin D_3_ concentrations of the diets were analyzed in aliquots of 1 g in triplicate. In the diets, the LLOQ for both vitamin D metabolites was 4.3 ng/g. Liver aliquots of 200 mg were analyzed for their concentrations of ergosterol, 7-DHC, vitamin D_2_, vitamin D_3_, 25(OH)D_2_, and 25(OH)D_3_. In the liver samples, the LLOQ was 5.0 ng/g for vitamin D_2_, 10.5 ng/g for vitamin D_3_, 0.3 ng/g for 25(OH)D_2_, and 2.1 ng/g for 25(OH)D_3_.

#### Analysis of Tocopherols in Plasma

To analyze the α-tocopherol concentrations in plasma, aliquots (30 μl) were mixed with pyrogallol solution (1% in ethanol, absolute) and saturated sodium hydroxide solution for hydrolysis. Subsequently, the samples were incubated at 70°C for 30 min, and tocopherols were extracted with *n*-hexane and ultrapure water. The supernatant was directly applied to the HPLC (26). HPLC conditions were the same as described for the tocopherol analysis of the oils.

#### Statistical Analysis

Data concerning the characterization of the plant oils were not subjected to statistical analysis. Values of the *in vivo* experiment are presented as means ± SD. If values were below the LLOQ, randomly generated values (between 0 and the appropriate LLOQ) were used for statistical treatment analyses. Statistical analyses were conducted using SPSS statistical software (SPSS 22, IBM; Armonk, NY, USA). All data were subjected to a normality test using the Shapiro–Wilk test. If the data followed a normal distribution, differences between the groups were analyzed by one-way analysis of variances (ANOVA), and subsequently subjected to the Levene’s test for homoscedasticity. In case of homogeneity of variance, the three treatment groups were compared by the Tukey’s test, in case of unequal variances by the Games–Howell test. If the data were not normal distributed, the Kruskal–Wallis test was used to analyze differences between the groups and the Mann–Whitney *U* test was conducted for *post hoc* comparisons of the three treatment groups (corrected by Bonferroni). Differences were considered to be significant at *P* < 0.05.

## Results

### Vitamin D and Vitamin D Precursors in Selected Plant Oils

**Figure 1:**
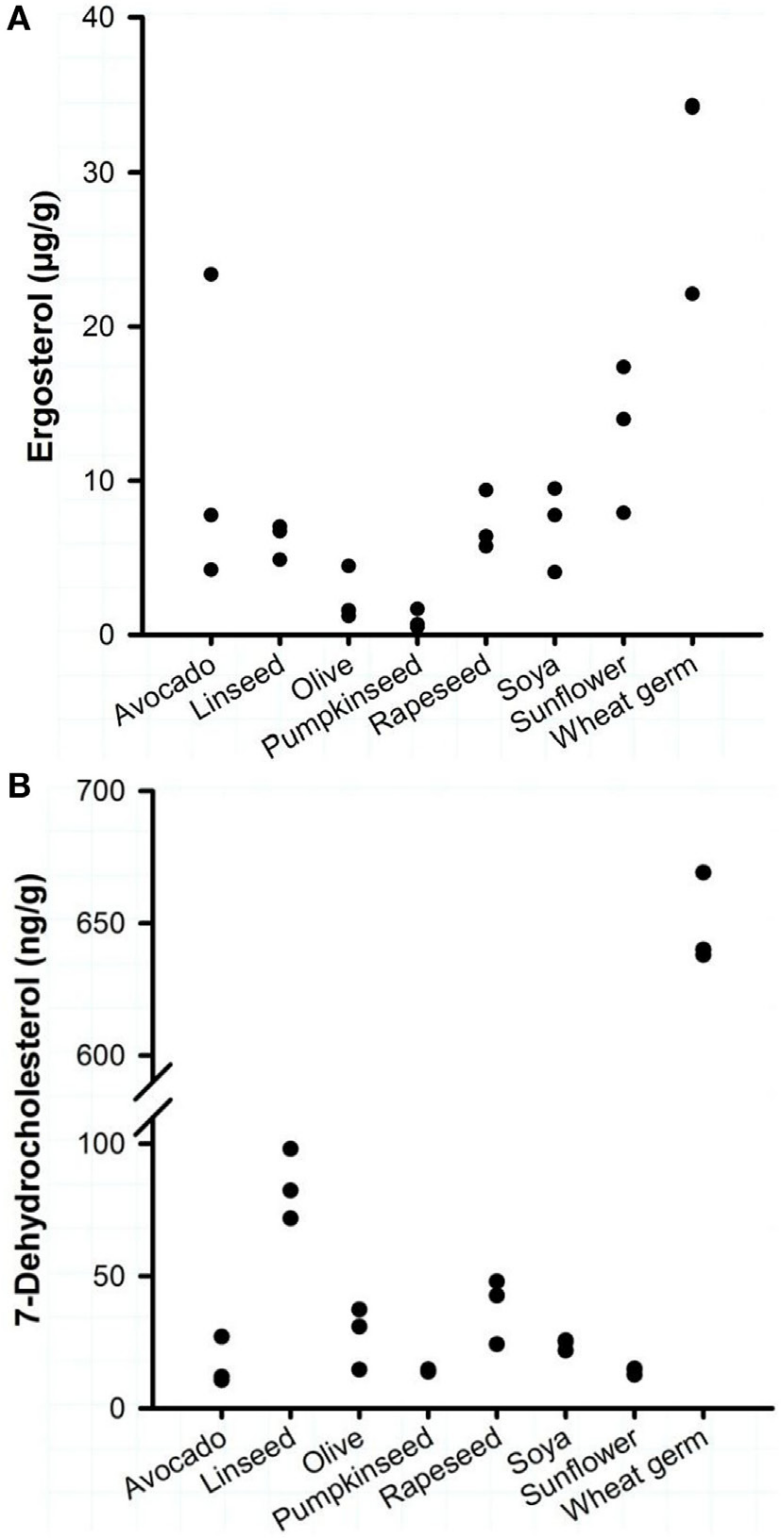
**Concentrations of (A) ergosterol and (B) 7-dehydrocholesterol of eight plant oils**. Each dot represents one oil from one manufacturer. Analyses were run in duplicate.

Eight commercially available plant oils for human nutrition were characterized for their vitamin D precursors and vitamin D contents. Analysis revealed that the concentrations of the vitamin D precursors ergosterol and 7-DHC varied strongly between the different oils, but all oils had a markedly higher concentration of ergosterol than of 7-DHC (Figure 1). The highest ergosterol concentration was found in the WGOs (22.1–34.2 μg/g) followed by the avocado oils (4.2–23.4 μg/g) and the sunflower oils (7.9–17.4 μg/g). Oils derived from rapeseed, soya, and linseed had lower ergosterol concentrations that ranged from 4.1 to 9.5 μg/g; the lowest concentrations were found in olive and pumpkinseed oils (<4.5 μg/g). Analyses revealed that the WGOs had the highest concentrations of 7-DHC (638–669 ng/g), while other oils had very low quantities of 7-DHC (Figure 1). The 7-DHC concentration in the linseed oils ranged between 71.7 and 97.5 ng/g; the other oils had 7-DHC concentrations between 10.7 and 47.9 ng/g. Vitamin D_2_ and D_3_ were not quantifiable in the eight analyzed plant oils.

#### Formation of Vitamin D in the UVB-Exposed Oils

**Figure 2:**
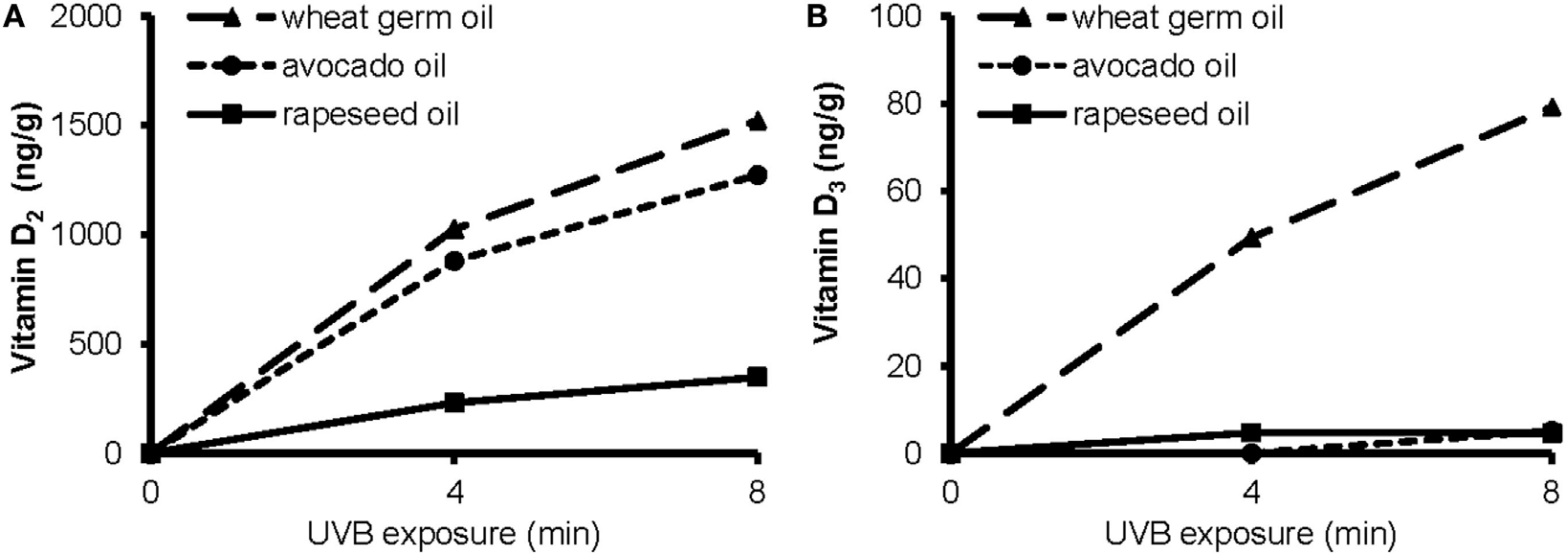
**Concentrations of (A) vitamin D_2_ and (B) vitamin D_3_ in wheat germ oil, avocado oil, and rapeseed oil before and after a 4 min- and an 8 min-UVB exposure**. UVB exposure conditions: oil layer thickness, 1.0 mm; UVB lamp distance, 10 cm.

To elucidate the impact of a short-term UVB irradiation on the formation of vitamin D in the plant oils, we exposed rapeseed oil, avocado oil, and WGO that differed widely in their amounts of vitamin D precursors to UVB light. The UVB exposure of rapeseed, avocado, and WGO increased the vitamin D concentrations in these oils in a time-dependent manner (Figure 2). The amount of vitamin D_2_ produced by UVB irradiation was higher in the wheat germ and the avocado oil than in the rapeseed oil. The amount of the vitamin D_3_ increased only in the WGO upon UVB exposure, but not in the rapeseed and the avocado oil, which was probably due to the higher 7-DHC concentration in the WGO (Figure 2).

The data further showed a significant impact of the layer thickness on the efficacy of the UVB exposure to increase the vitamin D content. The concentrations of vitamin D_2_ and vitamin D_3_ in the 1.6 mm-layer of WGO, which was UVB-exposed for 10 min, were 1035 and 37.0 ng/g, respectively. UVB-exposed WGO with a 3.2 mm-oil layer thickness had still high concentrations of vitamin D_2_ and vitamin D_3_, reaching 82 and 94% of the concentrations observed in the 1.6 mm-oil layer.

#### Changes in Quality Parameters of the Oils upon UVB Exposure

To elucidate the impact of the UVB treatment on the oil quality, the tocopherol concentrations and markers of autoxidation were measured in the UVB-exposed oils. The tocopherol concentrations of the 8-min UVB-exposed wheat germ and avocado oil (1.0 mm-layer) were not different from those of the untreated oils (Table 1). In the rapeseed oil, a slight decrease in the α- and γ-tocopherol contents upon UVB exposure was observed. A 10-min-UVB exposure of WGO (1.6 mm-layer) had again no effect on the tocopherol concentrations, and also the peroxide and the acid value were not affected (Table 2). Organoleptic analyses revealed that the UVB-exposed WGO had a slightly more off-flavor than the non-exposed oil sample of the same batch (Table 3).

**Table 1:** **Influence of UVB exposure^a^ on tocopherol concentrations of selected plant oils**.

Oil	UVB exposure^a^ time (min)	Tocopherols (mg/100 g)		
		α	β	γ
Rapeseed	0	19.1	0	36.8
	4	18.7	0	36.4
	8	16.8	0	36.0
Avocado	0	20.8	0.8	1.4
	4	20.0	0.6	1.3
	8	18.8	0.7	1.4
Wheat germ	0	140	51.1	14.0
	4	136	50.8	14.0
	8	136	51.5	11.3

*^a^UVB exposure conditions: oil layer thickness, 1.0 mm; UVB lamp distance, 10 cm*.

**Table 2:** **Influence of thermal treatment and storage on tocopherol concentrations, peroxide, and acid values of untreated and UVB-exposed^a^ wheat germ oil**.

Exposure	Treatment	Tocopherols (mg/100 g)			Peroxide value (mEq O_2_/kg)	Acid value (g KOH/kg)
		α	β	γ		
–	–	165	62.9	8.1	7.0	10.3
UVB	–	166	64.3	8.2	5.0	10.4
–	100°C, 10 min	164	63.2	8.3	8.8	9.8
UVB	100°C, 10 min	163	63.8	8.1	7.1	10.8
–	180°C, 10 min	159	59.3	7.7	1.0	10.2
UVB	180°C, 10 min	160	59.5	7.7	1.0	9.5
–	1 day storage (RT)	166	65.0	8.2	n. a.	n. a.
UVB	1 day storage (RT)	166	63.8	8.1	n. a.	n. a.
–	2 weeks storage (RT)	178	68.0	8.5	n. a.	n. a.
UVB	2 weeks storage (RT)	163	63.0	7.9	n. a.	n. a.
–	4 weeks storage (RT)	163	63.4	8.1	20.6	10.0
UVB	4 weeks storage (RT)	160	61.8	7.8	21.4	10.1

*^a^UVB exposure conditions: exposure time, 10 min; oil layer thickness, 1.6 mm; UVB lamp distance, 13 cm*.

*n. a., not analyzed; RT, room temperature*.

**Table 3:** **Influence of thermal treatment and storage on taste and aroma of UVB-exposed^a^ and non-exposed wheat germ oil**.

Exposure	Treatment	Taste^b^	Points^c^	Aroma^b^	Points^c^	Rank
–	–	Not quite neutral	4	Specific, neutral	5	1
UVB	–	Slightly rancid	3	Specific, neutral	5	2
–	100°C, 10 min	Slightly old	3	Specific, neutral	5	3
UVB	100°C, 10 min	Slightly old	3	Specific, neutral	5	4
–	180°C, 10 min	Rancid	2	Slightly dull, hay/seed	3	7
UVB	180°C, 10 min	Rancid, scratchy	1	Slightly dull, hay/seed	3	8
–	4 weeks storage (RT)	Slightly green	3	Specific, neutral	4	5
UVB	4 weeks storage (RT)	Slightly green	3	Specific, neutral	5	6

*^a^UVB exposure conditions: exposure time, 10 min; oil layer thickness, 1.6 mm; UVB lamp distance, 13 cm*.

*^b^The oil samples were tempered to 40°C*.

*^c^Correspondent: 1, poor; 2, insufficient; 3, satisfactory; 4, good; 5, excellent*.

*RT, room temperature*.

#### Susceptibility of UVB-Exposed Oils to Thermal Treatment and Storage

To elucidate the stability of a 10-min UVB-exposed WGO, we analyzed the concentrations of vitamin D metabolites and tocopherols, the peroxide and acid values, and the organoleptic quality after thermal treatment and after storage of the oil samples.

**Figure 3:**
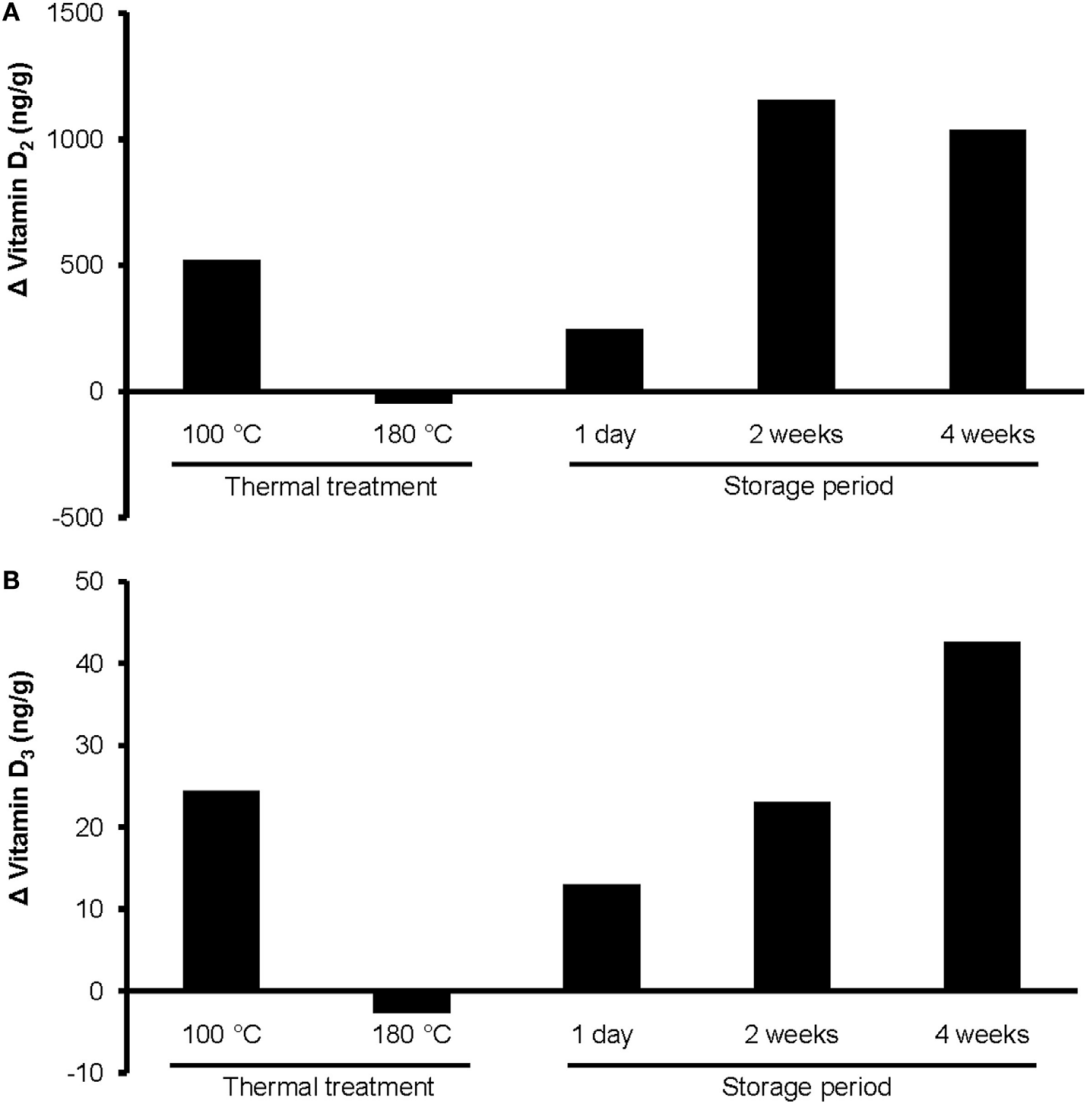
**Changes in the concentrations of (A) vitamin D_2_ and (B) vitamin D_3_ in UVB-exposed wheat germ oil after thermal treatment at 100 or 180°C for 10 min, and after a storage of 1 day, 2 weeks, or 4 weeks at room temperature in darkness**. UVB exposure conditions: exposure time, 10 min; oil layer thickness, 1.6 mm; UVB lamp distance, 13 cm. Analyses were run in duplicate.

The heating of UVB-exposed WGO at 100°C for 10 min resulted in a 50% increase of vitamin D_2_ (Δ = 521 ng/g) and a 66% increase in the vitamin D_3_ (Δ = 24.4 ng/g) concentration compared with the non-heated UVB-exposed WGO. In contrast, heating the oil at 180°C for 10 min resulted in a slight reduction of the vitamin D_2_ (Δ = −47.0 ng/g) and vitamin D_3_ (Δ = −2.8 ng/g) concentrations (Figure 3). Thermal treatment of the UVB-exposed and untreated WGO at 100°C had no effect on the analyzed markers for oxidation, while a thermal treatment at 180°C resulted in a slight reduction of the tocopherol concentrations and in a decrease in the peroxide value; the acid value remained unchanged (Table 2). Thermal treatment also affected the taste of the oil: the higher the treatment temperature, the lower was the organoleptic quality. The UVB exposure *per se* had only a small effect on the taste when the oil was heated at 180°C (Table 3).

The storage of UVB-exposed oil at room temperature also resulted in a rise of the vitamin D_2_ and the vitamin D_3_ concentrations (Figure 3). The highest vitamin D_2_ concentration was measured after the 2-week storage (Δ = 1157 ng/g; Figure 3A). The vitamin D_3_ concentration rose continuously during the 4-week storage and reached the highest values after 4 weeks (Figure 3B). The tocopherol concentrations in the UVB-exposed oil decreased slightly with the storage time; those of the untreated oil remained unchanged (Table 2). Both, the UVB-exposed and the -untreated oil showed increased peroxide values after the 4-week storage, no changes were observed for the acid values (Table 2). Organoleptic tests showed that the 4-week storage lead to deteriorated taste of the UVB-exposed and the -untreated oils, without showing any substantial difference between the UVB-exposed and the -untreated oil (Table 3). The aroma of the oils was not affected by the UVB exposure or the 4-week storage (Table 3).

### Efficacy of the UVB-Exposed Wheat Germ Oil to Improve the Vitamin D Status of Mice

To evaluate the efficacy of UVB-exposed WGO to improve the vitamin D status, a feeding study with mice was conducted. The analyzed concentrations of vitamin D precursors and vitamin D in the WGO demonstrate that the applied UVB treatment was capable of increasing the vitamin D_2_ and vitamin D_3_ in this oil (Table 4). The tocopherol concentrations in the untreated and the UVB-exposed oils were comparable (Table 4). Mice of the three groups did not differ in their daily food intake (WGO: 3.03 ± 0.23 g, WGO-UV: 3.01 ± 0.14 g, and WGO-D_3_: 3.11 ± 0.07 g) and final body mass (WGO: 31.5 ± 3.0 g, WGO-UV: 31.8 ± 2.9 g, and WGO-D_3_: 32.1 ± 1.6 g).

**Table 4:** **Characterization of the untreated and UVB-exposed wheat germ oils that were used in the mouse study**.

	Wheat germ oil	
	Untreated	UVB-exposed^a^
Ergosterol (μg/g)	42.2	42.3
7-DHC (ng/g)	960	921
Vitamin D_2_ (ng/g)	<LLOQ	850
Vitamin D_3_ (ng/g)	<LLOQ	34.8
Tocopherols (mg/g)		
α	1.32	1.34
β	0.51	0.50
γ	0.12	0.15

*^a^UVB exposure conditions: exposure time,10 min; oil layer thickness, 3.2 mm; distance of the UVB-emitting lamp, 13 cm*.

*7-DHC, 7-dehydrocholesterol; LLOQ, lower limit of quantification (4.3 ng/g)*.

**Figure 4:**
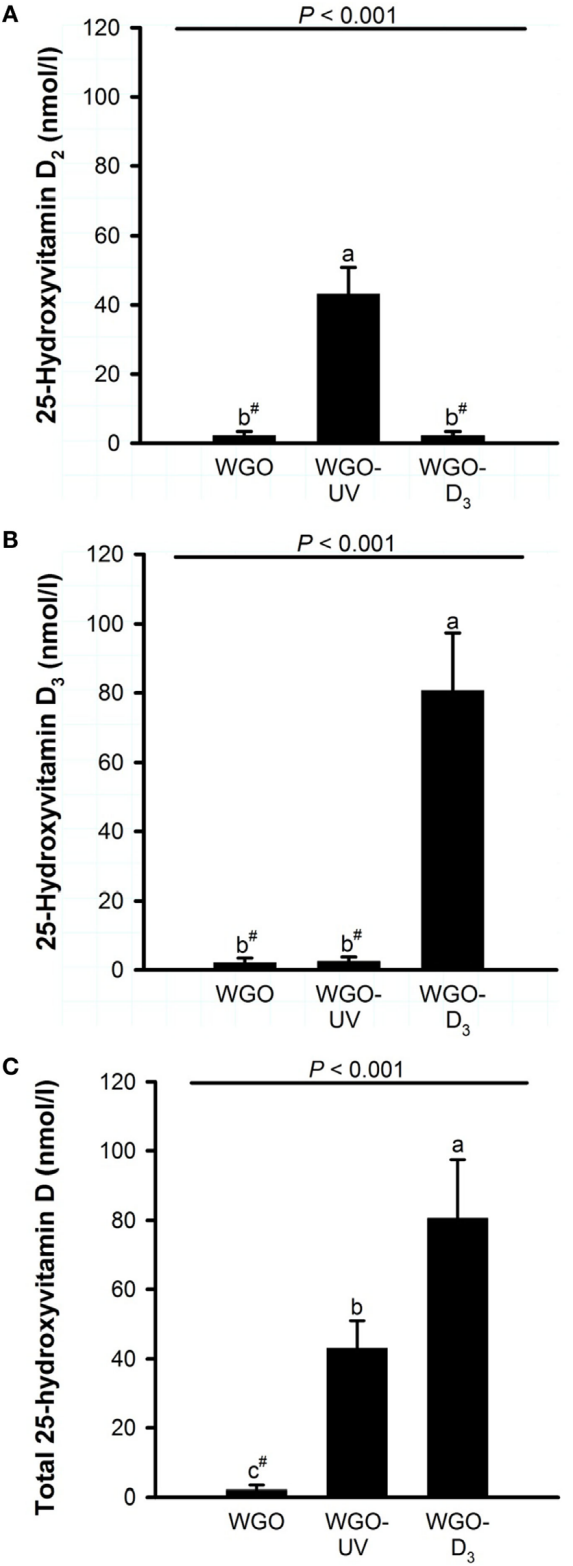
**Plasma concentrations of (A) 25-hydroxyvitamin D_2_, (B) 25-hydroxyvitamin D_3_, and (C) total 25-hydroxyvitamin D (25-hydroxyvitamin D_2_ + 25-hydroxyvitamin D_3_) of mice fed diets with 10% of either wheat germ oil (WGO), UVB-exposed wheat germ oil (WGO-UV), or wheat germ oil that was supplemented with vitamin D_3_ (WGO-D_3_) for 4 weeks**. Data represent means ± SD, *n* = 12. ^a–c^Means not sharing a letter are significantly different (*P* < 0.05, Mann–Whitney *U* test). ^#^Values were below the lower limit of quantification (4.2 nmol/l).

Because any changes in 25(OH)D upon feeding vitamin D are usually becoming the higher the lower the vitamin D status is at baseline (27), all mice received a vitamin D-free diet 5 weeks prior to the treatment with the UVB-exposed or vitamin D_3_-supplemented WGO. The plasma concentrations of total 25(OH)D [25(OH)D_2_ + 25(OH)D_3_] after feeding the vitamin D-free basal diet for 5 weeks was below the LLOQ (*n* = 6). Feeding mice the diet with UVB-exposed oil (WGO-UV) or with vitamin D_3_ supplemented oil (WGO-D_3_) for 4 weeks resulted in markedly higher plasma concentrations of total 25(OH)D compared with feeding the untreated WGO-based diet without any vitamin D supplementation (WGO) (*P* < 0.001). However, the increase in the total plasma concentration of 25(OH)D was stronger in the WGO-D_3_ group than in the WGO-UV group (*P* < 0.001). The predominant form of the plasma 25(OH)D in the WGO-UV group was 25(OH)D_2_; the predominant form in the WGO-D_3_ group was 25(OH)D_3_ (Figure 4). The plasma concentration of ergosterol was below the LLOQ in all groups of mice (Table 5). All mice had comparable plasma concentrations of 7-DHC. Mice from the WGO and WGO-D_3_ groups had plasma concentrations of vitamin D_2_ that were below the LLOQ, whereas mice from the WGO-UV group had more than 10-fold higher LLOQ values (Table 5). By contrast, the WGO-D_3_ group showed a markedly higher plasma concentration of vitamin D_3_ than the WGO-UV group (*P* < 0.001); the plasma concentration of vitamin D_3_ in the WGO group was below the LLOQ. No differences between the three groups were observed in the plasma concentrations of α-tocopherol (Table 5).

**Table 5:** **Plasma concentrations of D vitamers and α-tocopherol of mice fed diets with UVB-treated (WGO-UV) and vitamin D_3_-supplemented wheat germ oil (WGO-D_3_) compared with those fed the diet with non-treated wheat germ oil (WGO) for 4 weeks**.

Diet	WGO	WGO-UV	WGO-D_3_	*P* values
Ergosterol (nmol/l)	<LLOQ	<LLOQ	<LLOQ	–
7-DHC (nmol/l)	85.9 ± 18.8	83.3 ± 18.2	90.2 ± 28.8	n. s.
Vitamin D_2_ (nmol/l)	0.55^b,d^ ± 0.38	16.8^a^ ± 2.5	0.50^b,d^ ± 0.40	<0.001
Vitamin D_3_ (nmol/l)	0.36^c,d^ ± 0.28	1.44^b^ ± 1.34	20.5^a^ ± 3.9	<0.001
Total vitamin D (nmol/l)	0.55^c,d^ ± 0.38	16.8^b^ ± 2.5	20.5^a^ ± 3.9	<0.001
α-Tocopherol (μg/ml)	27.8 ± 3.8	29.9 ± 9.9	28.1 ± 5.9	n. s.

*Data represent means ± SD, n = 12*.

*WGO, 10% wheat germ oil; WGO-UV, 10% UVB-exposed wheat germ oil; WGO-D_3_, 10% wheat germ oil supplemented with vitamin D_3_; 7-DHC, 7-dehydrocholesterol; LLOQ, lower limit of quantification); n. s., not significant*.

*^a–c^Means not sharing a superscript letter are significantly different (*P* < 0.05, Mann–Whitney *U* test)*.

*^d^Values were below the LLOQ (1.25 nmol/l)*.

**Figure 5:**
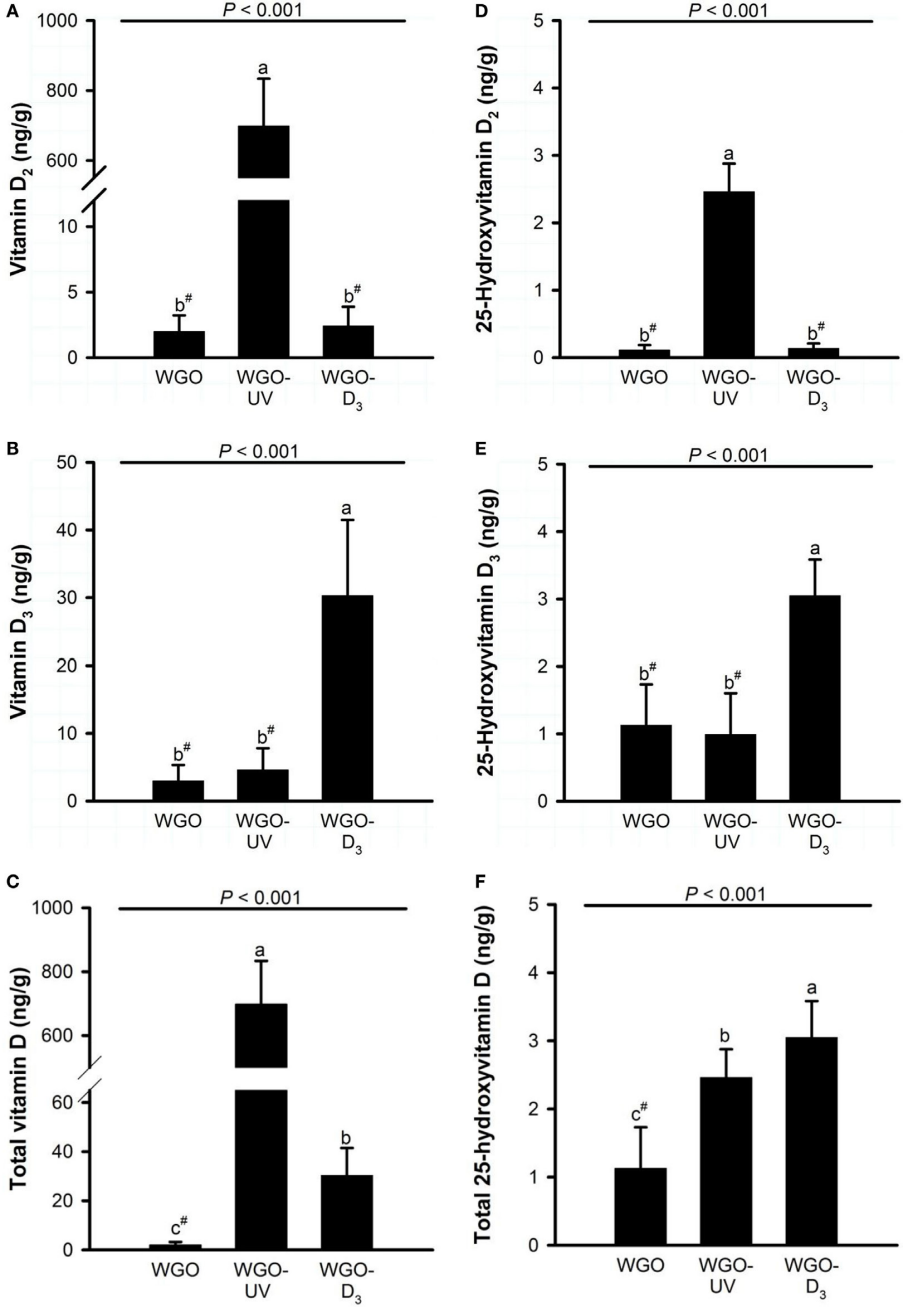
**Liver concentrations of (A) vitamin D_2_, (B) vitamin D_3_, (C) total vitamin D (vitamin D_2_ + vitamin D_3_), (D) 25-hydroxyvitamin D_2_, (E) 25-hydroxyvitamin D_3_, and (F) total 25-hydroxyvitamin D (25-hydroxyvitamin D_2_ + 25-hydroxyvitamin D_3_) in mice fed diets with 10% of either wheat germ oil (WGO), UVB-exposed wheat germ oil (WGO-UV) or wheat germ oil that was supplemented with vitamin D_3_ (WGO-D_3_) for 4 weeks**. Data represent means ± SD, *n* = 12. ^a–c^Means not sharing a letter are significantly different (*P* < 0.05, Mann–Whitney *U* test for vitamin D_2_, total vitamin D, 25-hydroxyvitamin D_2_, and 25-hydroxyvitamin D_3_; Games–Howell test for vitamin D_3_; Tukey test for total 25-hydroxyvitamin D). ^#^Values were below the lower limit of quantification (vitamin D_2_, 5.0 ng/g; vitamin D_3_, 10.5 ng/g; 25-hydroxyvitamin D_2_, 0.3 ng/g; 25-hydroxyvitamin D_3_, 2.1 ng/g).

Analysis of the D vitamer concentrations in the livers of the mice revealed no significant differences in the concentration of ergosterol (WGO: 12.0 ± 19.6 ng/g, WGO-UV: 3.92 ± 1.27 ng/g, and WGO-D_3_: 5.40 ± 2.73 ng/g) and 7-DHC (WGO: 94.3 ± 31.6 ng/g, WGO-UV: 84.7 ± 16.5 ng/g, and WGO-D_3_: 102 ± 46 ng/g). However, data showed distinct differences in the liver concentrations of vitamin D (Figure 5). Livers of mice from the WGO-UV group were characterized by extremely high vitamin D_2_ concentrations and high levels of 25(OH)D_2_, whereas the livers of mice from the WGO-D_3_ group had significantly higher vitamin D_3_ and 25(OH)D_3_ concentrations than those of mice from the two other groups (Figure 5).

## Discussion

The presented studies demonstrated that plant oils contain high amounts of ergosterol, but comparatively low amounts of 7-DHC. It was striking that the ergosterol concentrations in the plant oils were on average 100 times higher than the 7-DHC concentrations. It is assumed that plants are *per se* not capable of producing ergosterol or vitamin D_2_ (28), and that any of these metabolites are synthesized by endophytic fungi or by superficial fungal infections (13, 14, 29). Regarding 7-DHC, the analyses revealed 10 times higher concentration of this cholesterol precursor in the WGO than in the other oils. 7-DHC is an intermediate of the cholesterol synthesis pathway. It is well described that plants from the *Solanaceae, Fabaceae*, and *Poacaea* families are capable of producing cholesterol (30, 31), which is assumed to be used for the synthesis of glycoalkaloids and ecdysteroids (32, 33). The 7-DHC has also been proposed to function as an UV light protector (34), because the 7-DHC absorbs UVB irradiation that would otherwise damage the ribonucleic acids. The detectable amounts of 7-DHC in the linseed, rapeseed, and pumpkinseed oil suggest that cholesterol is also synthesized in plants from the *Linaceae, Brassicaceae*, and *Cucurbitaceae* families. However, in contrast to other researchers, who measured vitamin D in certain parts of the plant (12, 31, 35–37), we were not able to detect vitamin D in untreated plant oils.

The detection of vitamin D precursors in the plant oils prompted us to speculate that exposure of oils to UVB irradiation could convert ergosterol and 7-DHC into vitamin D_2_ and vitamin D_3_, respectively. Among the analyzed plant oils, the highest levels of vitamin D_2_ and vitamin D_3_ in response to an UVB irradiation were found in the WGO. After an 8-min exposure of thin-layered WGO, 1 g of this oil contained 1.5 μg vitamin D_2_ and 0.08 μg vitamin D_3_. We further found that the conversion rate of vitamin D precursors to vitamin D in the WGO was reduced by 40% if the oil layer thickness was increased from 1.0 to 3.2 mm. One gram of this thick-layered WGO provided in total a vitamin D content of 885 ng. With an average consumption of 12 g oil/day (38), a total of 10.6 μg vitamin D could be supplied by intake of UVB-exposed WGO, which matches 50% of the recommended daily vitamin D intake (1).

An interesting finding of this study was that the vitamin D content in the oils increased with the time of storage and a moderate thermal treatment. It is well described that the UVB photon converts the precursors, 7-DHC and ergosterol, to pre-vitamin D which in turn isomerizes to vitamin D by a thermal reaction (34, 39). Therefore, we assume that the preformed pre-vitamin D can convert to vitamin D in conditions with absent UVB irradiation. Our data further indicate that taste and aroma, and also biomarkers that are indicative of autoxidation such as the tocopherol concentration, peroxides, and free acids were not significantly influenced by a short-term exposure of the plant oils to UVB irradiation. This makes the short-term UVB treatment of plant oils to a safe and reliable technique to produce vitamin D supplements.

To evaluate the efficiency of UVB-exposed plant oils to improve the vitamin D status *in vivo*, we conducted a study with mice that were fed diets with either UVB-exposed WGO, untreated WGO, or WGO with supplemented vitamin D_3_. Here, we found that the UVB-exposed WGO is suitable to improve the vitamin D status of the mice as the group fed the UVB-exposed oil developed higher 25(OH)D plasma levels than the group fed the untreated oil. Compared with the group fed the vitamin D_3_-supplemented WGO, the UVB-exposed oil was less effective in increasing the 25(OH)D plasma concentrations. However, it should be noted that the livers of mice that received the UVB-exposed WGO stored huge amounts of vitamin D_2_ in comparison to that of mice fed the vitamin D_3_ supplemented oil. The increased storage of hepatic vitamin D_2_ in combination with the reduced plasma concentration of 25(OH)D_2_ in the group fed the UVB-exposed oil suggests that vitamin D_2_ is less appropriate as a substrate for hepatic hydroxylation than vitamin D_3_. It has been a debate for many years whether both forms of vitamin D are bioequivalent. A series of studies has shown that vitamin D_2_ does not increase 25(OH)D serum concentrations to the same amount as vitamin D_3_ does (40–42). The current data confirm the different efficacy of both vitamin D isoforms. However, we cannot exclude at this stage, that photo-isomers that are produced by the UVB treatment may also impact the bioavailability of the vitamin D form in UVB-exposed oil.

To conclude, plant oils that are commonly used in human nutrition contain considerable quantities of ergosterol, but small amounts of 7-DHC. Among the different analyzed oils, WGO has the highest amounts of vitamin D precursors. A short-term UVB irradiation was successful in increasing the vitamin D content of the selected oils. The *in vivo* study has shown that UVB-exposed WGO can improve the vitamin D status, although less effective than vitamin D_3_.

## Author Contributions

CB, BK, and GS conceived and designed the experiment. AB performed the experiment. AB and FH analyzed the data. AB, CB, and GS wrote the manuscript. BK and FH critically reviewed the manuscript.

## Conflict of Interest Statement

The authors declare that the research was conducted in the absence of any commercial or financial relationships that could be construed as a potential conflict of interest.

## Abbreviations

25(OH)D, 25-hydroxyvitamin D; 7-DHC, 7-dehydrocholesterol; LLOQ, lower limit of quantification; WGO, wheat germ oil.

## References

[1] Ross AC Manson JE Abrams SA Aloia JF Brannon PM Clinton SK The 2011 report on dietary reference intakes for calcium and vitamin D from the Institute of Medicine: what clinicians need to know. J Clin Endocrinol Metab (2011) 96:53–8. 10.1210/jc.2010-2704 21118827 PMC3046611

[2] Brown J Sandmann A Ignatius A Amling M Barvencik F. New perspectives on vitamin D food fortification based on a modeling of 25(OH)D concentrations. Nutr J (2013) 12:151. 10.1186/1475-2891-12-151 24261676 PMC3874620

[3] Lehmann U Rosendahl Gjessing H Hirche F Mueller-Belecke A Gudbransen OA Magne Ueland P Efficacy of fish intake on vitamin D status: a meta-analysis of randomized controlled trials. Am J Clin Nutr (2015) 102:837–47. 10.3945/ajcn.114.105395 26354531

[4] Ko JA Lee BH Lee JS Park HJ. Effect of UV-B exposure on the concentration of vitamin D2 in sliced shiitake mushroom (*Lentinus edodes*) and white button mushroom (*Agaricus bisporus*). J Agric Food Chem (2008) 56:3671–4. 10.1021/jf073398s 18442245

[5] European Food Safety Authority. Scientific opinion on the safety of vitamin D-enriched UV-treated baker’s yeast1: EFSA panel on dietetic products, Nutrition and Allergies (NDA). EFSA J (2014) 12:3520 10.2903/j.efsa.2014.3520

[6] Haussler MR Hughes MR McCain TA Zerwekh JE Brumbaugh PF Jubiz W 1,25-Dihydroxyvitamin D_3_: mode of action in intestine and parathyroid glands, assay in humans and isolation of its glycoside from *Solanum malacoxylon*. Calcif Tissue Res (1977) 22:1–18. 10.1007/BF02064033 912510

[7] Boland R Skliar M Curino A Milanesi L Vitamin D compounds in plants. Plant Sci (2003) 164:357–69. 10.1016/S0168-9452(02)00420

[8] Zimmerman DR Koszewski NJ Hoy DA Goff JP Horst RL. Targeted delivery of 1,25-dihydroxyvitamin D3 to colon tissue and identification of a major 1,25-dihydroxyvitamin D3 glycoside from *Solanum glaucophyllum* plant leaves. J Steroid Biochem Mol Biol (2015) 148:318–25. 10.1016/j.jsbmb.2014.10.019 25445916 PMC4361337

[9] Mello J Calcinosis – calcinogenic plants. Toxicon (2003) 41:1–12. 10.1016/S0041-0101(02)00241-6 12467655

[10] Rambeck W Oesterhelt W Vecchi M Zucker H Occurrence of cholecalciferol in the calcinogenic plant *Trisetum flavescens*. Biochem Biophys Res Commun (1979) 87:743–9. 10.1016/0006-291X(79)92021-7 222271

[11] Horst RL Reinhardt TA Russel JR Napoli JL. The isolation and identification of vitamin D2 and vitamin D3 from *Medicago sativa* (alfalfa plant). Arch Biochem Biophys (1984) 231:67–71. 10.1016/0003-9861(84)90363-1 6326678

[12] Aburjai T Al-Khalil S Abuirjeie M Vitamin D3 and its metabolites in tomato, potato, egg plant and zucchini leaves. Phytochemistry (1998) 49:2497–9. 10.1016/S0031-9422(98)00246-5

[13] Graham JH Abbott LK Wheat responses to aggressive and non-aggressive arbuscular mycorrhizal fungi. Plant Soil (2000) 220:207–18. 10.1023/A:1004709209009

[14] Li H Smith SE Holloway RE Zhu Y Smith FA. Arbuscular mycorrhizal fungi contribute to phosphorus uptake by wheat grown in a phosphorus-fixing soil even in the absence of positive growth responses. New Phytol (2006) 172:536–43. 10.1111/j.1469-8137.2006.01846 17083683

[15] Ohyama K Suzukia M Kikuchi J Saito K Muranaka T. Dual biosynthetic pathways to phytosterol via cycloartenol and lanosterol in *Arabidopsis*. Proc Natl Acad Sci U S A (2009) 106:725–30. 10.1073/pnas.0807675106 19139393 PMC2621255

[16] Kühn J Hirche F Geissler S Stangl GI. Oral intake of 7-dehydrocholesterol increases vitamin D_3_ concentrations in the liver and kidney. J Steroid Biochem Mol Biol (2015). 10.1016/j.jsbmb.2015.12.017 26709139

[17] Mattila PH Piironen VI Uusi-Rauva EJ Koivistoinen PE Contents of cholecalciferol, ergocalciferol, and their 25-hydroxylated metabolites in milk products and raw meat and liver as determined by HPLC. J Agric Food Chem (1995) 43:2394–9. 10.1021/jf00057a015

[18] Higashi T Shibayama Y Fuji M Shimada K. Liquid chromatography-tandem mass spectrometric method for the determination of salivary 25-hydroxyvitamin D3: a noninvasive tool for the assessment of vitamin D status. Anal Bioanal Chem (2008) 391:229–38. 10.1007/s00216-007-1780-3 18087693

[19] Coors U Anwendung des Tocopherolmusters zur Erkennung von Fett- und Ölvermischungen. Fat Sci Technol (1991) 93:519–26.

[20] Deutsche Gesellschaft für Fettwissenschaften e.V. Säurezahl, 81. Stuttgart: Wissenschaftliche Verlagsgesellschaft mbH (1994).

[21] Amtliche Sammlung von Untersuchungsverfahren nach § 64 LFGB. Bestimmung der Peroxidzahl in tierischen und pflanzlichen Fetten und Ölen, L13.0037. Amtliche Sammlung von Untersuchungsverfahren. Berlin: Beuth Verlag GmbH (2012).

[22] Deutsche Gesellschaft für Fettwissenschaften e.V. Äußere Baschaffenheit – Sensorische Prüfungen, 14. Stuttgart: Wissenschaftliche Verlagsgesellschaft mbH (2014).

[23] Amtliche Sammlung von Untersuchungsverfahren nach § 64 LFGB. Rangordnungsprüfung – Sensorische Prüfverfahren, L00.904. Amtliche Sammlung von Untersuchungsverfahren. Berlin: Beuth Verlag GmbH (2011).

[24] National Research Council. Guide for the Care and Use of Laboratory Animals. Washington, DC: National Academies Press (2011).

[25] Reeves PG Nielsen FH Fahey GC. AIN-93 Purified diets for laboratory rodents: final report of the American Institute of Nutrition ad hoc writing committee on the reformulation of the AIN-76A rodent diet. J Nutr (1993) 123:1939–51. 8229312 10.1093/jn/123.11.1939

[26] Eder K Skufca P Brandsch C. Thermally oxidized dietary fats increase plasma thyroxine concentrations in rats irrespective of the vitamin E and selenium supply. J Nutr (2002) 132:1275–81. 12042446 10.1093/jn/132.6.1275

[27] Lehmann U Riedel A Hirche F Brandsch C Girndt M Ulrich C Vitamin D3 supplementation: response and predictors of vitamin D3 metabolites – a randomized controlled trial. Clin Nutr (2016) 35(2):351–8. 10.1016/j.clnu.2015.04.021 26037521

[28] Seitz LM Sauer B Burroughs R Mohr HE Hubbard JD Ergosterol as a measure of fungal growth. Phytopathology (1979) 69:1202–3. 10.1094/Phyto-69-1202

[29] Saif SR Khan AG The effect of vesicular-arbuscular mycorrhizal associations on growth of cereals: III. Effects on barley growth. Plant Soil (1977) 47:17–26. 10.1007/BF00010364

[30] Moreau RA Whitaker BD Hicks KB. Phytosterols, phytostanols, and their conjugates in foods: structural diversity, quantitative analysis, and health-promoting uses. Prog Lipid Res (2002) 41:457–500. 10.1016/S0163-7827(02)00006-1 12169300

[31] Jäpelt RB Silvestro D Smedsgaard J Jensen PE Jakobsen J LC-MS/MS with atmospheric pressure chemical ionisation to study the effect of UV treatment on the formation of vitamin D3 and sterols in plants. Food Chem (2011) 129:217–25. 10.1016/j.foodchem.2011.04.029

[32] Bergenstråhle A Borgå P Jonsson LM Sterol composition and synthesis in potato tuber discs in relation to glycoalkaloid synthesis. Phytochemistry (1996) 41:155–61. 10.1016/0031-9422(95)00554-4

[33] Dinan L. Phytoecdysteroids: biological aspects. Phytochemistry (2001) 57:325–39. 10.1016/S0031-9422(01)00078-4 11393511

[34] Björn LO Wang T Is provitamin D a UV-B receptor in plants? Plant Ecol (2001) 154:1–8. 10.1023/A:1012985924283

[35] Prema TP Raghuramulu N. Vitamin D3 and its metabolites in the tomato plant. Phytochemistry (1996) 42:617–20. 10.1016/0031-9422(95)00883-7 8768317

[36] Skliar M Curino A Milanesi L Benassati S Boland R *Nicotiana glauca*: another plant species containing vitamin D3 metabolites. Plant Sci (2000) 156:193–9. 10.1016/S0168-9452(00)00254-5 10936526

[37] Jäpelt RB Silvestro D Smedsgaard J Jensen PE Jakobsen J. Quantification of vitamin D3 and its hydroxylated metabolites in waxy leaf nightshade (*Solanum glaucophyllum* Desf.), tomato (*Solanum lycopersicum* L.) and bell pepper (*Capsicum annuum* L.). Food Chem (2013) 138:1206–11. 10.1016/j.foodchem.2012.11.064 23411232

[38] Max-Rubner-Institut, editor. Nationale Verzehrsstudie II: Ergebnisbericht Teil 2. Karlsruhe: Die bundesweite Befragung zur Ernährung von Jugendlichen und Erwachsenen (2008). Available from: https://www.bmel.de/SharedDocs/Downloads/Ernaehrung/NVS_ErgebnisberichtTeil2.pdf

[39] Wacker M Holick MF Sunlight and vitamin D: a global perspective for health. Dermatoendocrinol (2013) 5:51–108. 10.4161/derm.24476 24494042 PMC3897598

[40] Trang HM Cole DE Rubin LA Pierratos A Siu S Vieth R. Evidence that vitamin D3 increases serum 25-hydroxyvitamin D more efficiently than does vitamin D2. Am J Clin Nutr (1998) 68:854–8. 9771862 10.1093/ajcn/68.4.854

[41] Romagnoli E Mascia ML Cipriani C Fassino V Mazzei F D’Erasmo E Short and long-term variations in serum calciotropic hormones after a single very large dose of ergocalciferol (vitamin D2) or cholecalciferol (vitamin D3) in the elderly. J Clin Endocrinol Metab (2008) 93:3015–20. 10.1210/jc.2008-0350 18492750

[42] Heaney RP Recker RR Grote J Horst RL Armas LA. Vitamin D(3) is more potent than vitamin D(2) in humans. J Clin Endocrinol Metab (2011) 96:E447–52. 10.1210/jc.2010-2230 21177785

